# Darier’s disease: treatment with topical sodium diclofenac 3% gel^☆^

**DOI:** 10.1016/j.abd.2022.01.019

**Published:** 2023-06-29

**Authors:** Marcella Oliveira Menezes Quitete de Campos, Giovanna Abrantes Pimenta de Figueiredo, Allyson Capobiango Evangelista, Alexander Richard Bauk

**Affiliations:** Department of Dermatology, Hospital Federal dos Servidores do Estado do Rio de Janeiro, Rio de Janeiro, RJ, Brazil

Dear Editor,

A 32-year-old male patient came to the authors outpatient clinic with a previous history of epilepsy and cognitive impairment, using carbamazepine and a clinical and histopathological diagnosis of Darier's disease for eight years. On physical examination, he had multiple erythematous-brown, keratotic papules, some of them crusted, located on the dorsal region, shoulders and anterior thorax (Fig. 1). He had previously used topical keratolytics, corticosteroids and retinoids, in addition to systemic antibiotics, without achieving lesion control. Systemic retinoids were avoided due to possible drug interactions (carbamazepine).

**Figure 1:**
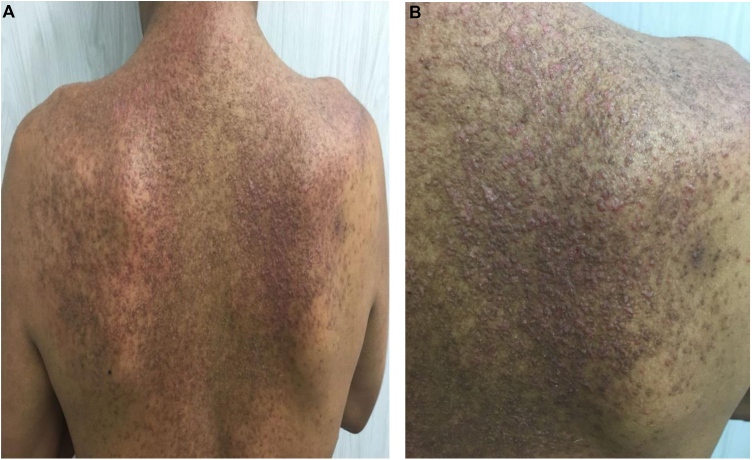
(A) Dorsal region ‒ before treatment. (B) Right scapular region, before treatment

After reviewing the literature, the authors found some reports on the use of topical sodium diclofenac in Darier's disease. They chose to start with 3% sodium diclofenac and 2.5% hyaluronic acid in natrosol gel, applied twice a day only on the affected areas on the left side of the body, for eight weeks. After significant improvement of the lesions, the patient was instructed to apply it on all affected areas, twice a day. He denied local or systemic adverse reactions during medication use and no laboratory changes were observed during this period. After four months of treatment, he showed significant lesion regression, with post-inflammatory hypo- and hyperchromic macules and the presence of a few residual keratotic papules (Fig. 2). Medication use was reduced to once a day for another four weeks and the treatment was discontinued, with good clinical control maintained since then.

**Figure 2:**
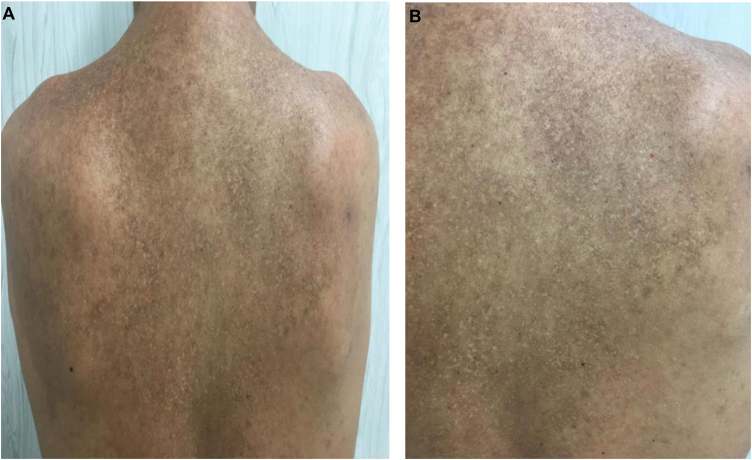
(A) Dorsal region, four months after starting treatment. (B) Detail of the dorsal region, right scapular region after treatment

Darier's disease occurs due to a mutation in the ATP2A2 gene, which encodes a protein involved in epidermal differentiation and intercellular communication called SERCA2B, leading to impaired SERCA21 function.^1^, ^2^ It manifests as erythematous-brown, keratotic-crusted papules, in seborrheic areas of the trunk, scalp, face, and neck associated with a foul odor and pruritus.^1^ Ungual changes may also be observed, such as erythematous and white longitudinal bands, longitudinal fissures, subungual keratosis, and brittleness, forming “V”-shaped notches.^1^

Treatment of Darier's disease includes general measures such as wearing light clothing and sunscreen protection; use of topical medications, such as keratolytics, corticoids, retinoids, tacrolimus, and 5-fluorouracil; and systemic drugs such as acitretin, isotretinoin, or cyclosporine. Surgical and photodynamic therapy can also be performed.^1^

Sodium diclofenac 3% was recently described as a therapeutic option in Darier's disease.^2^, ^3^, ^4^ Its mechanism of action occurs by inhibiting cyclooxygenase-2, resulting in the suppression of prostaglandin E2 activity, which in turn downregulates the ATP2A2 gene. This leads to the normalization of SERCA2 levels in keratinocytes.^1^ Its use is combined with hyaluronic acid 2.5%, which acts by maintaining the drug on the epidermis and superficial dermis, with better local action and less systemic absorption.^5^

## Financial support

None declared.

## Authors' contributions

Marcella Oliveira Menezes Quitete de Campos: Drafting and editing of the manuscript; critical review of the literature.

Giovanna Abrantes Pimenta de Figueiredo: Drafting and editing of the manuscript; critical review of the literature.

Allyson Capobiango Evangelista: Drafting and editing of the manuscript; critical review of the literature.

Alexander Richard Bauk: Approval of the final version of the manuscript; design and planning of the case report; effective participation in research orientation; intellectual participation in the propaedeutic and/or therapeutic conduct of the studied cases; critical review of the literature; critical review of the manuscript.

## Conflicts of interest

None declared.

## References

[1] Kamijo M., Nishiyama C., Takagi A., Nakano N., Hara M., Ikeda S. (2011). Cyclooxygenase-2 inhibition restores ultraviolet B-induced downregulation of ATP2A2/SERC2 in keratinocytes: possible therapeutic approach of cyclooxygenase-2 inhibition for treatment of Darier disease. Br J Dermatol..

[2] Millan-Parrilla F., Rodrigo-Nicolas B., Moles-Poveda P., Armengot-Carbo M., Quecedo-Estebanez E., Gimeno-Carpio E. (2014). Improvement of Darier disease with diclofenac sodium 3% gel. J Am Acad Dermatol..

[3] Santos-Alarcon S., Sanchis-Sanchez C., Mateu-Puchades A. (2016). Diclofenac sodium 3% gel for Darier’s disease treatment. Dermatol Online J..

[4] Palacios-Alvarez I., Andres-Ramos I., Silva M.Y., Simal G. (2017). Treatment of Darier’s disease with diclofenac sodium 3% gel. Dermatol Ther..

[5] Nelson C.G. (2011). Diclofenac gel in the treatment of actinic keratosis. Ther Clin Risk Manag..

